# Bromodomain protein Brd3 promotes *Ifnb1* transcription via enhancing IRF3/p300 complex formation and recruitment to *Ifnb1* promoter in macrophages

**DOI:** 10.1038/srep39986

**Published:** 2017-01-03

**Authors:** Wenhui Ren, Chunmei Wang, Qinlan Wang, Dezhi Zhao, Kai Zhao, Donghao Sun, Xingguang Liu, Chaofeng Han, Jin Hou, Xia Li, Qian Zhang, Xuetao Cao, Nan Li

**Affiliations:** 1 National Key Laboratory of Medical Immunology and Institute of Immunology, Second Military Medical University, Shanghai 200433, China; 2 Institute of Basic Medical Sciences, Chinese Academy of Medical Sciences, School of Basic Medicine Peking Union Medical College, Beijing 100005, China; 3 Institute of Immunology, Zhejiang University School of Medicine, Hangzhou 310058, Zhejiang, China

## Abstract

As members of bromodomain and extra-terminal motif protein family, bromodomain-containing proteins regulate a wide range of biological processes including protein scaffolding, mitosis, cell cycle progression and transcriptional regulation. The function of these bromodomain proteins (Brds) in innate immune response has been reported but the role of Brd3 remains unclear. Here we find that virus infection significantly downregulate Brd3 expression in macrophages and Brd3 knockout inhibits virus-triggered IFN-β production. Brd3 interacts with both IRF3 and p300, increases p300-mediated acetylation of IRF3, and enhances the association of IRF3 with p300 upon virus infection. Importantly, Brd3 promotes the recruitment of IRF3/p300 complex to the promoter of *Ifnb1*, and increases the acetylation of histone3/histone4 within the *Ifnb1* promoter, leading to the enhancement of type I interferon production. Therefore, our work indicated that Brd3 may act as a coactivator in IRF3/p300 transcriptional activation of *Ifnb1* and provided new epigenetic mechanistic insight into the efficient activation of the innate immune response.

Innate immune response is the first defense line in hosts to fight against pathogens. It can be initiated by the pattern recognition receptors and sensors in immune cells and then transduce signals to produce inflammatory cytokines and Type I interferon, IFN-β. When infected with viruses, IFN-β is the mostly produced cytokine which is very powerful and has important consequences in anti-virus response^1^,^2^. Multiple molecules are involved in the regulation of this process, such as IRF3, a crucial transcription factor which can fine tune the production of IFN-β^3^. Despite major advances in our understanding of cellular regulation and signaling pathways of Type I interferon induction, the components of the pathways and the epigenetic regulators involved have not been fully elucidated.

Bromodomain protein 3 (Brd3) is a member of the bromodomain and extra-terminal motif protein (BET) family which includes four members Brd2, Brd3, Brd4 and Brdt^4^. The name of BETs comes from the protein domains the family members consist of: two bromodomains and an extra terminal domain. Bromodomain is the sole protein module for recognition of acetylated lysine^5^. Many transcriptional regulation proteins such as the transcription co-activators GCN5, P/CAF, p300/CBP contain bromodomain^6^. The extra terminal domain of BETs has been found to interact with specific effector proteins and recruit them to regulate target gene transcription^7^. The BET proteins have been demonstrated as protein scaffolds, mitotic bookmarks, cell cycle regulators and transcription regulators^8^,^9^,^10^,^11^,^12^,^13^. Among the BET family proteins, Brd2 and Brd3 are the most closely related members^4^. The coupling of histone acetylation to transcription *in vivo* by Brd2 and Brd3 has been demonstrated^14^. Both Brd2 and Brd3 were capable of allowing transcription in the absence of factor FACT(facilitates chromatin transcription), suggesting that they possess histone chaperone activity^14^. However, these two proteins are not simply redundant. Except the interaction with histones, Brd3 could also combine with transcription factors, such as GATA1 and promote its chromatin occupancy at erythroid target genes^15^. Brd4 has been found acting as a co-activator for the transcriptional activation of NF-κB^16^, suggesting that BETs might participate in immune response^17^,^18^,^19^.

In our previous effort to identify molecules selectively involved in the regulation of innate immune response against viral infection^20^, we found Brd3 decreased nearly 2 folds after VSV infection in macrophages by genome-wide screening. Together with the data mining results of the GEO profiles that reveals Brd3 downregulation after various virus infection (see Results), these evidences strongly suggested that Brd3 may be involved in the process of virus-triggered immune response. In this study, the function of Brd3 in virus-initiated immune response was addressed. We demonstrated that Brd3 is an indispensable molecule for macrophages to produce IFN-β after virus infection. It can interact with IRF3/p300 complex, and enhances their recruitment to the *Ifnb1* promoter after viral infection. We further demonstrate that Brd3 increases the acetylated histone3/histone4 within the *Ifnb1* promoter. Therefore, our work revealed Brd3 as a positive regulator in the production of IFN-β in response to viral infection, and provided new mechanistic insight into the efficient activation of the innate immune response.

## Results

### Virus infection down regulates Brd3 expression in macrophages

**Figure 1:**
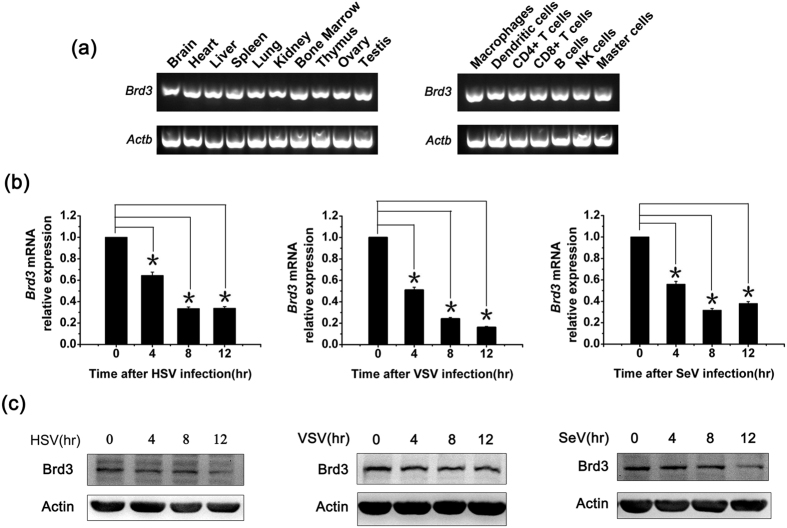
Virus infection down-regulates Brd3 expression in macrophages. (**a**), Total RNA was extracted from different mouse tissues and immune cells, 1 μg RNA was used to perform reverse transcription-PCR. Real time-PCR was performed to analysis the *Brd3* mRNA expression level, *Actb* was used as a control. Then the reaction product was analysed by agarose electrophoresis. (**b**), Mouse peritoneal macrophages were infected with HSV (MOI = 10), VSV (MOI = 10), SeV (MOI = 10) for the indicated times. The *Brd3* mRNA expression level was detected by Q-PCR. The results were presented as fold expression of *Brd3* mRNA to that of *Actb*. (**c**), Mouse peritoneal macrophages were treated as in b, cells were lysed and subjected to Western blot analysis(40 μg) with the indicated antibodies. Data are representative of three independent experiments with similar results (**a** and **c**), and data are shown as means ± SD of three independent experiments. *P < 0.05(analysis of variance (ANOVA)).

We first examined the expression pattern of Brd3 in mouse normal tissues and immune cells by RT-PCR. As shown in Fig. 1a, Brd3 was ubiquitously expressed in various mouse tissues, including immune organs such as the thymus, bone marrow, and spleen. Further detection of Brd3 expression in immune cells revealed that Brd3 was also expressed in various immune cells including macrophages and NK cells (Fig. 1a).

Our systemic analysis identified several genes of BET family that were downregulated significantly in macrophages in response to VSV infection, including Brd3^20^. Data mining of Brd3 expression pattern in NCBI GEO profiles also revealed similar data including measles virus brain infection model in mice (GDS4553/1450902_at/Brd3), pandemic H1N1 influenza virus infections of human bronchial epithelial cells (GDS4855/203825_at/BRD3), and treatment with Hepatitis C virus core protein of the hepatocyte cell line (GDS2239/212547_at/BRD3), indicating the decreased expression of Brd3 after various virus infection. We then wondered whether various virus infection would affect the expression of Brd3 in macrophages. Primary peritoneal macrophages were treated with HSV, VSV and SeV for different times, respectively. The result showed that Brd3 mRNA levels significantly decreased in virus-stimulated macrophages (Fig. 1b). Virus infection also markedly down regulated Brd3 protein expression in a time-dependent manner (Fig. 1c). These data indicate that Brd3 might be involved in virus triggered immune response.

### Brd3 is indispensable for the production of IFN-β in virus-infected macrophages

**Figure 2:**
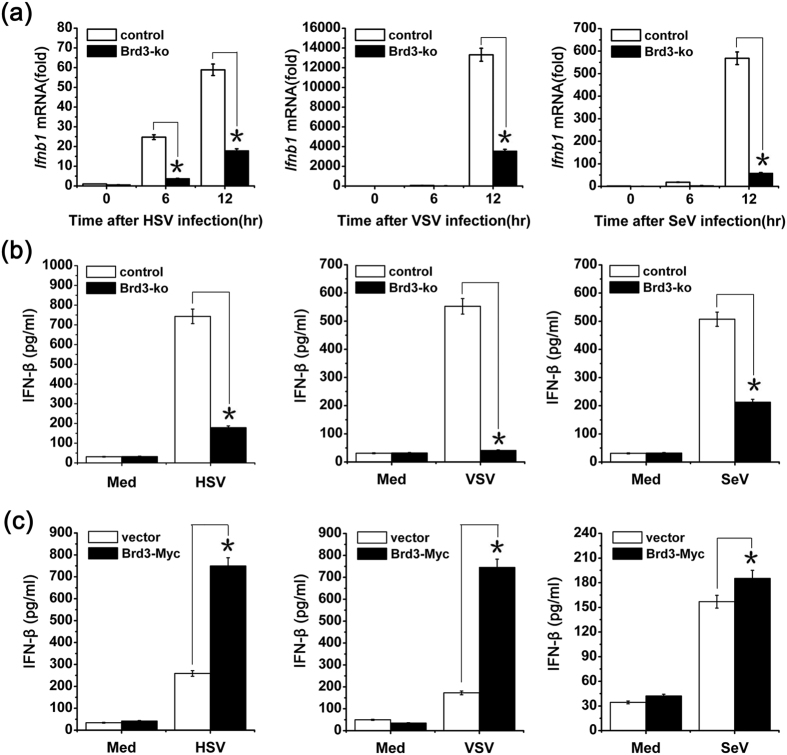
Brd3 is indispensable for the production of IFN-β in virus-infected RAW264.7 cells. (**a**), Brd3-ko cells and control cells were infected with HSV (MOI = 10), VSV (MOI = 10), SeV (MOI = 10) for the indicated times. The *Ifnb1* mRNA level was detected by Q-PCR. The results were presented as fold expression of *Ifnb1* mRNA to that of *Actb*. (**b**), Brd3-ko cells and control cells were infected with HSV (MOI = 10), VSV (MOI = 10), SeV(MOI = 10) for 12 hours or left unstimulated (Med), and then the production of IFN-β in the supernatants was measured by ELISA. (**c**), Brd3-ko cells were transiently transfected with a Brd3 expression plasmid (Brd3-Myc) or empty vector, 24 hours later, cells were infected with HSV(MOI = 10), VSV(MOI = 10), SeV(MOI = 10) for 12 hours or left unstimulated (Med), and then the production of IFN-β in the supernatants was measured by ELISA. Data are shown as means ± SD of three independent experiments. *P < 0.05(Student’s *t*-test).

To investigate the role of Brd3 in virus-induced innate immune response, CRISPR-Cas9 technology was used to knockout Brd3 expression in RAW264.7 cells (Supplementary Fig. 1a). Then we used this cell line to examine the production of pro-inflammatory cytokines and type I interferon triggered by virus infection. The results showed that, after virus infection Brd3 knockout (Brd3-ko) cells showed significantly decreased IFN-β production both in mRNA and protein levels (Fig. 2a,b), and, to a much lesser extent, IL-6 production (Supplementary Fig. 1b). However, the production of pro-inflammatory cytokine TNF-α was not affected (Supplementary Fig. 1c). Different clones of Brd3-ko cells showed similar results (Supplementary Fig. 2a–c) and increased virus replicates (Supplementary Fig. 2d). The results in primary murine macrophages further confirmed that Brd3 has a positive role in IFN-β production (Supplementary Fig. 3). To confirm the increased production of IFN-β mediated by Brd3, Brd3-ko cells were transfected with Myc-tagged Brd3 expression vector for 24 hours (Supplementary Fig. 4) and then infected with viruses. As shown in Fig. 2c, the virus-induced production of IFN-β was restored by reintroducing Brd3 expression into Brd3-ko cells. These results indicate that Brd3 can promote IFN-β expression in virus activated macrophages.

Furthermore, we evaluated the effect of Brd3 on the phenotype of macrophages. A Flow cytometry analysis of macrophages transfected with specific Brd3 si-RNA showed that the expression of CD11b and F4/80 was barely affected by Brd3 knocked down in macrophages before or after VSV infection(data not shown), indicating that Brd3 has no influence on macrophage phenotype.

### Brd3 associates with IRF3 and promotes IRF3-mediated IFN-β production

**Figure 3:**
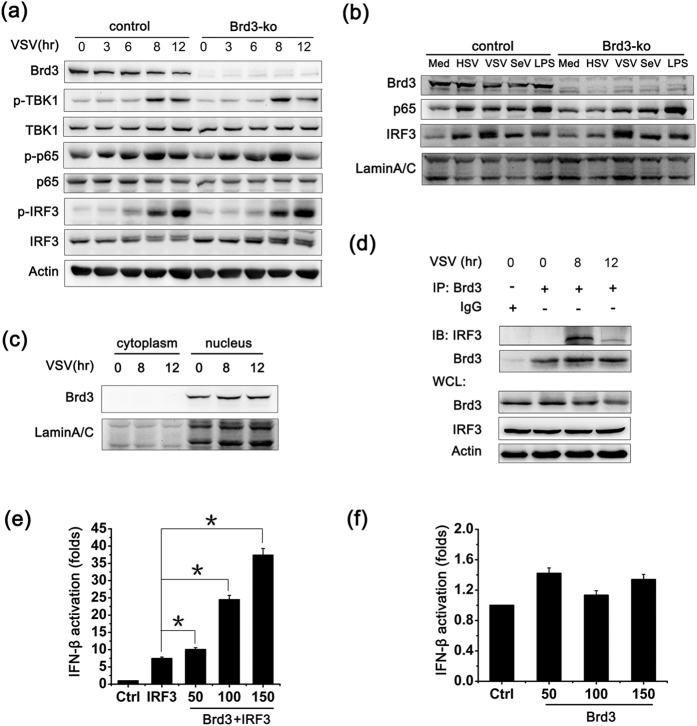
Brd3 associates with IRF3 and promotes IRF3-mediated IFN-β production. (**a**), Brd3-ko cells and control cells were infected with VSV(MOI = 10) for the indicated times. Cells were lysed and subjected to Western blot analysis (40 μg) with the indicated antibodies. (**b**), Brd3-ko cells and control cells were infected with HSV (MOI = 10), VSV (MOI = 10), SeV (MOI = 10) for 8 hours or stimulated with LPS(100 ng/ml) for 90 minutes. Nuclear proteins were extracted and subjected to Western blot analysi s(25 μg) with the indicated antibodies. (**c**), RAW264.7 cells were infected with VSV (MOI = 10) for the indicated times. Cells were extracted as cytoplasmic and nuclear proteins and subjected to Western blot analysis (25 μg) with the indicated antibodies. (**d**), RAW264.7 cells were infected with VSV (MOI = 10) for the indicated times. Cell lysates (500 μg) were immunoprecipitated with Brd3 antibody and then immunoblotted with the indicated antibodies. Similar results were obtained in three independent experiments. (**e**), HEK293T cells were co-transfected with 100 ng IRF3-wide type expressing plasmid; 50 ng IFN-β luciferase reporter plasmid; and 5 ng pTK-*Renilla*-luciferase reporter plasmid together with 50, 100, or 150 ng of Brd3-expressing plasmid. Total amounts of plasmid DNA were equalized using an empty control vector. After 24 hours of culture, luciferase activity was measured and normalized by *Renilla* luciferase activity. (**f**), HEK293T cells were co-transfected with 50 ng IFN-β luciferase reporter plasmid; 5 ng pTK-*Renilla*-luciferase reporter plasmid together with 50, 100, or 150 ng of Brd3-expressing plasmid and detected as in (**e**). Data are shown as means ± SD of three independent experiments. *P < 0.05(ANOVA).

We then explored the underlying mechanism for increasing virus-induced IFN-β expression by Brd3. Virus-triggered signaling, especially the TBK1-IRF3 signal pathway which is important for the production of IFN-β were under first consideration. As shown in Fig. 3a, Brd3 knockout hardly had any effect on VSV-triggered phosphorylation of TBK1, p65 or IRF3. And the translocation of p65 and IRF3 into the nucleus was not affected either by Brd3 knockout after virus infection or LPS challenge (Fig. 3b). It’s been reported that Brd3 mainly resides in nucleus^21^. We also confirmed the predominant nuclear localization of Brd3 in Raw 264.7 macrophages (Fig. 3c), indicating that Brd3 may mainly function in nucleus in macrophages, to regulate IFN-β transcription. The multiple functional BETs can interact with transcription factors^16^. Given the important role of IRF3 in the production of type I interferon^22^, we investigated whether Brd3 positively regulated IFN-β production by interacting with IRF3. Immunoprecipitation assays with lysates from RAW 264.7 cells that express Brd3 and IRF3 endogenously were performed to test whether these two proteins can interact with each other. As shown in Fig. 3d, IRF3 could be detected in Brd3 immunoprecipitates and the interaction was increased after virus infection. To further investigate the effect of Brd3 association with IRF3, Myc-tagged Brd3 expression vector were co-transfected with Flag-tagged IRF3 expression vector and IFN-β luciferase reporter plasmid in HEK293T cells to detect the luciferase activity of IFN-β by dual-luciferase reporter assay. The results showed that IRF3-mediated reporter activity of IFN-β was significantly enhanced by Brd3 in a dose-dependent manner (Fig. 3e), whereas Brd3 overexpression alone couldn’t affect the IFN-β reporter activity (Fig. 3f). These results suggest that Brd3 could associate with IRF3 in nucleus and promote IRF3-mediated IFN-β activation.

### Brd3 enhances p300-mediated acetylation of IRF3

**Figure 4:**
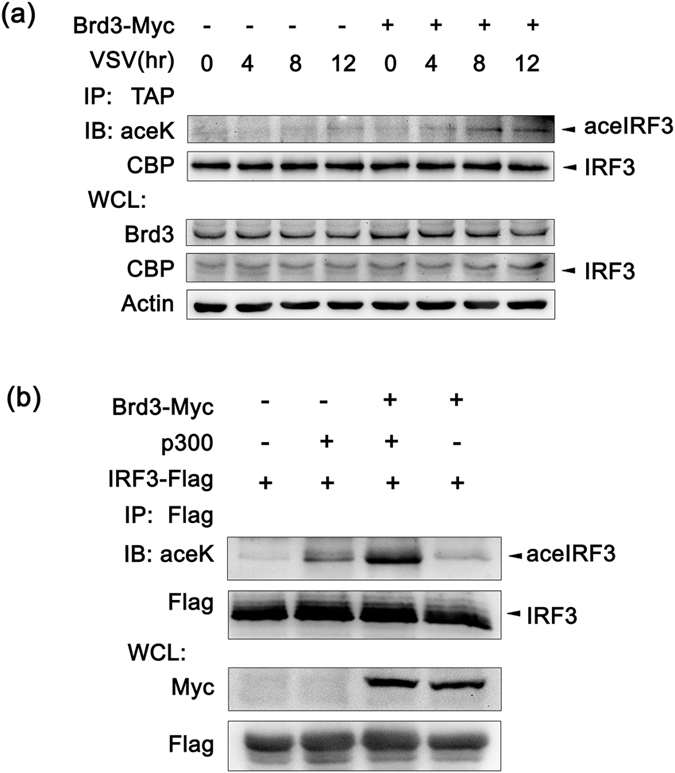
Brd3 promotes p300 mediated acetylation of IRF3. (**a**), RAW 264.7 cells stably overexpressing TAP-tagged IRF3 were transiently transfected with Brd3-Myc plasmid or empty vector for 36 hours and then infected with VSV(MOI = 10) for the indicated times. Cell lysates (500 μg) were immunoprecipitated with Dynabeads which can bind with TAP tag and then immunoblotted with the indicated antibodies. (**b**), HEK293T cells were co-transfected with IRF3-Flag, Brd3-Myc and p300 expressing plasmid for 48 hours and then infected with VSV (MOI = 0.01) for 8 hours. Cell lysates (500 μg) were immunoprecipitated with anti-Flag antibody and then immunoblotted with the indicated antibodies. Similar results were obtained in three independent experiments.

Besides being phosphorylated which is required for the activation, IRFs could also be acetylated (26,27) although their acetylation has not been well studied. As the bromodomains in Brd3 has the ability to recognize acetylated proteins, we then examined the acetylation status of IRF3 after virus infection. The result showed that overexpression of Brd3 increased the acetylation level of IRF3 after VSV infection (Fig. 4a). It is reported that acetyltransferase p300 may have the ability to acetylate IRF3^23^,^24^. We further co-transfected Flag-tagged IRF3, p300 and Myc-tagged Brd3 expression vectors into 293T cells. 48 hours later cells were infected with VSV for 8 hours and then subjected to IP and then Western blot analysis. As shown in Fig. 4b, the acetylation level of IRF3 was increased when p300 were co-expressed, and this modification has been reinforced when Brd3 was overexpressed. However, overexpression of Brd3 alone had minimal effect on the acetylation level of IRF3. These results suggest that Brd3 can promote p300-mediated acetylation of IRF3.

### Brd3 associates with p300 and enhances the interaction between IRF3 and p300

**Figure 5:**
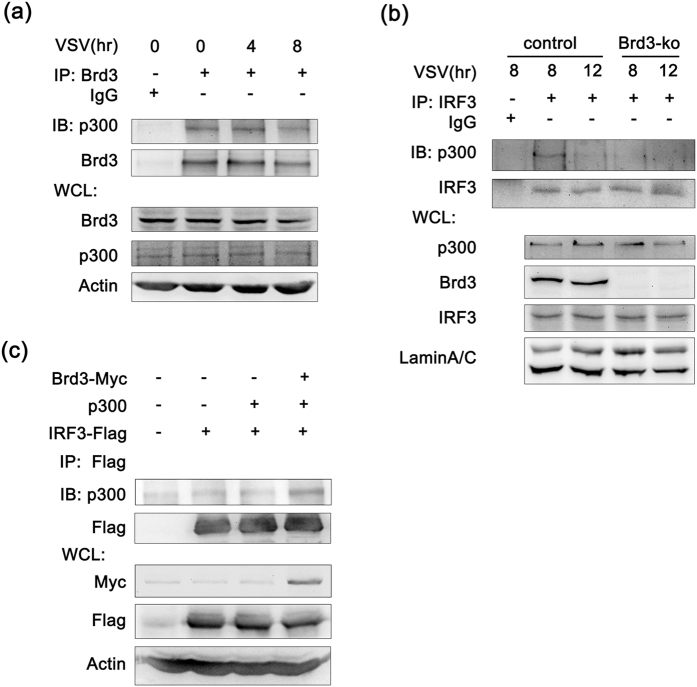
Brd3 associates with p300 and enhances the interaction between IRF3 and p300. (**a**), RAW264.7 cells were infected with VSV(MOI = 10) for the indicated times. Cell lysates (500 μg) were immunoprecipitated with Brd3 antibody and then immunoblotted with the indicated antibodies. (**b**), Brd3-ko cells and control cells were infected with VSV (MOI = 10) for 8 hours. Nuclear proteins were extracted and immunoprecipitated (250 μg) with IRF3 antibody and then immunoblotted with the indicated antibodies. (**c**), HEK293T cells were co-transfected with IRF3-Flag, Brd3-Myc and p300 expressing plasmid. After 48 hours, cell lysates (250 μg) were immunoprecipitated with anti-Flag antibody and then immunoblotted with the indicated antibodies. Similar results were obtained in three independent experiments.

After being phosphorylated in the cytoplasm, IRF3 would translocate into the nucleus and interact with p300 to initiate the transcription of the type I IFN and ISG genes^22^. Although IRF3 displays relatively strong DNA binding activity, its transactivation appears to be dependent on p300 which can acetylate histones and facilitate the transcription process. Considering that Brd3 could interact with IRF3 and enhance p300-mediated acetylation of IRF3, we speculated that Brd3 could also be involved in the formation of IRF3/p300 complex. Co-IP experiments showed that endogenous p300 could be detected in Brd3 immunoprecipitants and the association could also be detected in unstimulated RAW264.7 cells (Fig. 5a). We further investigated the role of Brd3 in binding with IRF3/p300 complex. For endogenous interaction of IRF3 and p300, co-IP analysis were performed in lysates of Brd3-ko and control cells, and result showed that the interaction between IRF3 and p300 was attenuated in Brd3-ko cells compared with control cells after VSV infection (Fig. 5b). Furthermore, we transfected Flag-tagged IRF3, p300 and Myc-tagged Brd3 expression vectors into 293T cells and immunoprecipitated Flag for Western blot analysis. As shown in Fig. 5c, binding of p300 and IRF3 in 293T cells was markedly enhanced by Brd3 overexpression. Together, these experiments provide evidence that Brd3 interacts with IRF3/p300 complex and promotes the association between IRF3 and p300, suggesting that they may act as a complex to co-regulate gene transcription.

### Brd3 recruits IRF3/p300 complex to the promoter of *Ifnb1* and facilitates the transcription of *Ifnb1*

**Figure 6:**
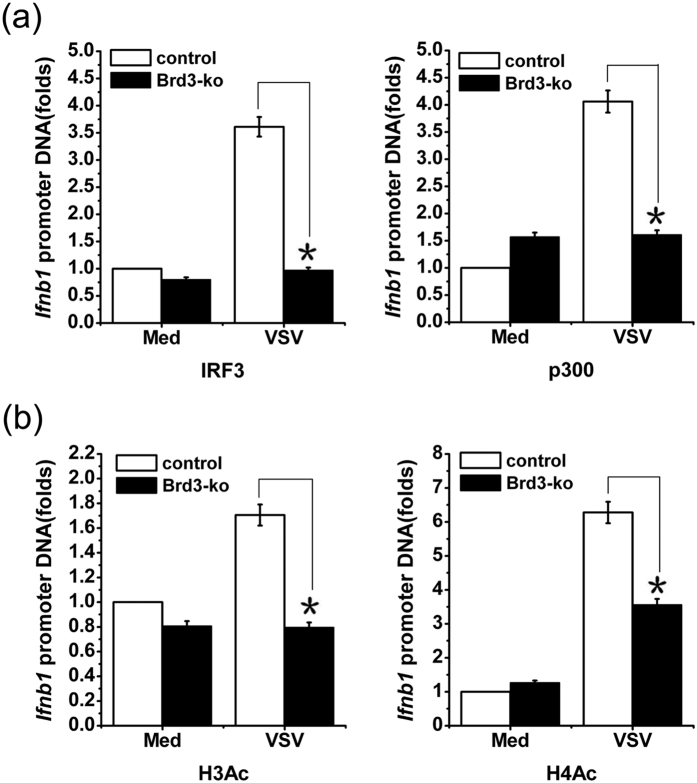
Brd3 recruits IRF3/p300 complex to the promoter of *Ifnb1* and facilitates the transcription of *Ifnb1*. (**a**), Brd3-ko cells and control cells were infected with VSV(MOI = 10) for 8 hours or left unstimulated (Med). The recruitment of IRF3 and p300 to the *Ifnb1* gene promoter was detected with a ChIP assay and analyzed by Q-PCR. (**b**), Brd3-ko cells and control cells were infected with VSV (MOI = 10) for 8 hours or left unstimulated (Med). The amount of acetylated histone3 and histone4 within *Ifnb1* gene promoter was detected with a ChIP assay and analyzed by Q-PCR. Data are shown as means ± SD of three independent experiments. *P < 0.05(Student’s *t*-test).

Our observation that Brd3 increased virus-induced IFN-β production independent of NF-κB and TBK1-IRF3 signaling pathways implied that Brd3 may mediate gene-specific transcription regulation in gene locus of *Ifnb1*. To this end, ChIP assay was performed to evaluate the effect of Brd3 on IRF3 and p300 recruitment to the *Ifnb1* promoter. As shown in Fig. 6a, virus-induced binding of IRF3 and p300 to the gene promoter of *Ifnb1* decreased significantly in Brd3-ko cells, suggesting that Brd3 enhances virus-triggered IRF3 and p300 recruitment to *Ifnb1* promoter.

As acetyl-lysine “readers”, Brd2 and Brd3 have been found to couple histone acetylation to transcription. We did indeed detect Brd3 association with acetylated histone3 and histone4, even in unstimulated macrophages (Supplementary Fig. 5). Given the fact that histone acetylation is implicated in regulating transcription of type I interferon during innate immune response, we therefore tested whether histone acetylation at the *Ifnb1* promoter was affected in Brd3-ko cells after VSV infection. The result showed that, when Brd3 protein has been knocked out, the acetylated histone3 and histone4 within *Ifnb1* promoter was reduced significantly comparing with control cells after virus infection (Fig. 6b). However, the acetylated histone3 and histone4 within *Tnfa* promoter showed no difference in Brd3-ko cells and control cells (Supplementary Fig. 6). Taken together, these results indicate that Brd3 promotes IRF3/p300 transcription activity by enhancing virus-triggered IRF3/p300 recruitment to *Ifnb1* promoter and assisting the acetylation of histone3 and histone4, leading to the promotion of type I interferon production.

## Discussion

IFN-β expression is dependent on the assembly of a transcription enhanceosome consisting of many transcription factors. IRF3 is one of the main transcription factors responsible for recruiting the histone acetyltransferases p300/CBP to the *Ifnb1* enhanceosome^3^. In this work, we demonstrated that Brd3, as epigenetic regulators, has functionally important consequences for antiviral responses. Our results have shown that the virus-induced interaction of IRF3 and p300 was attenuated in Brd3-ko macrophages. Notably, knockout of Brd3 inhibited VSV-induced recruitment of both IRF3 and p300 to the *Ifnb1* promoter. These data support the hypothesis that Brd3 associates with the IFN-β enhanceosome by interacting with IRF3 and p300 and enhances their recruitment to the *Ifnb1* promoter to increase histone hyperacetylation and transcription of *Ifnb1* in virus-triggered innate immune responses. Given that knockout of Brd3 inhibited the phosphorylation of neither TBK1 nor IRF3 and p65 and impaired recruitment of IRF3/p300 to the *Ifnb1* promoter, we propose that Brd3 mediate virus–induced production of type I interferon by acting as a coactivating pathway for such interferon production.

A lot of observations have underscored the importance of the transcription factor IRF3 and the coactivator p300 in immune response against viral infection^3^,^25^. Our study demonstrated that Brd3 could positively regulate IRF3/p300 complex transcriptional activation toward virus-induced IFN-β production by interacting with and enhancing the formation of IRF3/p300 complex. As acety-lysine “readers”, bromodomain-containing proteins recognize and decode the acetylated proteins to regulate their activities. Therefore, the increased Brd3 interaction with IRF3/p300 complex upon viral infection we detected here might possibly correlated with the acetylation status of either IRF3 or p300 (or both). It’s been shown that although their acetylation has not been well studied, IRFs could also be acetylated^23^,^24^. The coactivator and acetyltransferase p300 may be among the potential acetyltransferases responsible for the IRF3 acetylation. Suhara *et al*. has demonstrated that IRF3 could be acetylated by p300 by *in vitro* experiments^23^,^24^. Here we found that Brd3 increased the acetylation level of IRF3 after VSV infection (Fig. 4a). When co-expressed with IRF3 and p300 in 293T cells, Brd3 promoted p300-mediated acetylation of IRF3 (Fig. 4b). Therefore, while Brd3 contains no HAT domain, it may promote the acetylation process by recruiting other acetyltransferase (e.g., p300) to substrate proteins like IRF3. Further experiments to determine whether Brd3 (or other bromodomain proteins) interacts with acetylated IRF3 via specific bromodomain and to identify the precise sites of IRF3 acetylation as well as a sequence motif that is specifically recognized by the bromodomains would be helpful to reveal the detailed mechanisms underlining the interaction of Brd3 with IRF3/p300 complex.

In this work, we found that Brd3 could interact with IRF3, but not transcription factor NF-κB (data not shown). However, there’s report demonstrated that Brd4 could interact with NF-κB via the binding of the bromodomain to the acetylated-Lys^310^ residue on the RelA subunit^16^. The differences may partially due to the sequence flexibilities and structural diversities among BETs^21^,^26^. Whether Brd4 could interact with NF-κB in the macrophage context remains to be established. The differentially regulation of transcription by different family members reflects the nonredudancy in the functions of bromodomain-containing proteins. This underscores the need for systematic genome-wide approaches to catalog genes associated and regulated by BETs, which will facilitate to describe the physiological and pathological roles of these proteins.

As a positive regulator of IFN-β transcription activation, Brd3 expression was downregulated after virus infection, as demonstrated by genome-wide screening, data mining of the GEO profiles, as well as mRNA and protein level analysis of virus-infected macrophages (Fig. 1). This is different from other activators found participating in the production of IFN-β, which might be a fine-tuning mechanism possessed by the host to prevent the superfluous IFN-β production. Proper downregulation of the expression of pro-inflammatory cytokines is necessary for the host to avoid excessive cytotoxicity and tissue injury after infection. Diverse mechanisms in signal transduction^27^ and PTM level^20^,^22^ exist to allow well-timed attenuation of inflammatory reactions. The multiplicity of feedback regulation indicates the presence of divergent and maybe combined mechanisms that altogether assure timely cessation of cytokine induction. Brd3 enhancement of IRF3/p300 recruitment to the gene promoter may contribute to the collective inhibition by introducing a distinct mechanism that exerts at the level of *Ifnb1* transcription.

Owing to the rapid development of both biological function and structural basis of BET proteins, the inhibitors of BETs have become a newly emerging therapeutic strategy for cancer and other diseases^8^,^28^. Collectively, our results raise the possibility that Brd3 expression and regulation has important consequences for Type I interferon responses and targeting Brd3 specifically may have benefits in viral infection.

## Methods

### Mice, Antibodies and Reagents

C57BL/6 mice (6 to 8 weeks) were obtained from Joint Ventures Sipper BK Experimental Animal Co., Shanghai, China. All animal experiments were performed in accordance with the National Institute of Health Guide for the Care and Use of Laboratory Animals, with the approval of the Scientific Investigation Board of Second Military Medical University, Shanghai. Antibodies against phosphorylated TBK1, TBK1, phosphorylated p65(Ser-536), p65, phosphorylated IRF3, IRF3, LaminA/C, Myc tag and Flag tag were from Cell Signaling Technology. Antibodies against Brd3 and Actin were from Sigma-Aldrich. Antibody against acetyl-lysine was obtained from Abcam. Antibodies against p300, acetyl-Histone3, acetyl-Histone4 and calmodulin binding protein (CBP) epitope tag were obtained from Millipore. Dynabeads MyOne Streptavidin C1 was from Invitrogen. HSV was from Prof. Q. Li(Chinese Academy of Sciences, Beijing, China), VSV was a gift from Prof. W. Pan (Second Military Medical University, Shanghai, China), and Sendai virus (SeV) was from Prof. B. Sun (Chinese Academy of Sciences, Shanghai, China).

### Plasmid Constructs

A recombinant expression vector encoding Myc-tagged Brd3 was constructed by PCR cloning into the pReceiver-M43 eukaryotic expression vector (Fulengene Genecopoeia). The pRL-TK-*Renilla*-luciferase and IFN-β luciferase reporter plasmids were described previously^29^. The p300 and Flag-tagged IRF3 expression vector was constructed by PCR cloning into the pcDNA3.1 eukaryotic expression vector (Invitrogen). All constructs were confirmed by DNA sequencing.

### Cell culture, and transfection

Thioglycolate-elicited mouse primary peritoneal macrophages were isolated and cultured as described previously^30^. Mouse macrophage cell line RAW 264.7 and human HEK293T cells were obtained from the American Type Culture Collection (Manassas, VA) and cultured as described^31^. RAW 264.7 cells stably overexpressing TAP-tagged IRF3 vectors which contains a calmodulin binding protein (CBP) epitope in TAP tag was established in our lab. For transient transfection of plasmids in HEK293T cells, jetPEI reagent was used according to manufacturer’s instructions (Polyplus).

### RNA Interference

The specific siRNA for Brd3 (si-Brd3) and control siRNA (si-Non) were purchased from Gene Pharma and delivered into mouse peritoneal macrophages using INTERFERin reagent (Polyplus) as described previously^32^. si-Brd3 were synthesized as follows: 5′-GGAGAGCUCUUCAGAUUCATT-3′(sense), and 5′-UGAAUCUGAAGAGCUCUCCTT-3′ (anti-sense).

### Phenotype analysis

Cells were incubated for 15 min at 4 °C with fluorescein-conjugated monoclonal Abs in labeling solution. Fluorescein-conjugated isotype controls were used to establish the background. Flow cytometry analyses were conducted on LSR II (BD Biosciences). Data were analyzed with CELLQuest or FACSDiva software (BD Biosciences) as described previously^33^.

### CRISPR-Cas9-mediated gene knockout

CAS9/green fluorescent protein and Brd3 guide RNA plasmids (Shanghai Biomodel Organism Science & Technology Development Co. Ltd) were transiently transfected into RAW 264.7 cells using jetPEI. Single transfected cells were sorted into individual wells in a 96-well plate using the MoFlo XDP, expanded, and screened by immunoblot.

### Real-time Quantitative PCR

The extraction of RNA and operation of Real-time quantitative PCR were performed as described previously^32^. The primers used for *Brd3* were 5′-AACCCTCCAGACCACGAAGT-3′ (sense) and 5′-GCACAGAGGAGACATTCAACAG-3′ (anti-sense). Primers used for VSV were: 5′-ACGGCGTACTTCCAGATGG-3′ (sense) and 5′-CTCGGTTCAAGATCCAGGT-3′ (anti-sense). Primers used for *Actb* were 5′-AGTGTGACGTTGACATCCGT-3′ (sense) and 5′-GCAGCTCAGTAACAGTCCGC-3′ (anti-sense). Data were normalized by the level of *Actb* expression in each sample.

### ELISA

IFN-β, TNF-α and IL-6 levels in the supernatants were measured by ELISA kit (R&D) according to the manufacturer’s instructions.

### Immunoblot and immunoprecipitation

The nuclear and cytoplasmic extracts were prepared with NE-PER nuclear and cytoplasmic extraction reagents (Pierce). Immunoblot and immunoprecipitation were performed as described previously^31^.

### Luciferase Reporter Gene Assay

HEK293T cells were cotransfected with a mixture of the IFN-β luciferase reporter plasmid, pRL-TK-*Renilla*-luciferase plasmid, and the appropriate additional constructs for 24 hours. Total amounts of DNA were equalized with empty control vector. Luciferase activities were measured using dual-luciferase reporter assay system (Promega) as described previously^32^. Data were normalized for transfection efficiency by comparing firefly luciferase activity with that of *Renilla* luciferase.

### ChIP assay

Chromatin was immunoprecipitated using anti-IRF3, p300, acetyl-Histone3 or acetyl-Histone4 antibody as described previously^32^. The primers of the promoter of *Ifnb1* were: 5′-AGAGACCCTCTCCCACCATC-3′ (sense), 5′-ATTGCTGGAGCAAAGGAAGA-3′(anti-sense). The primers of the promoter of were: 5′-ACAGAATCCTGGTGGGGACGACGGG-3′ (sense), 5′- CAGACGGCCGCCTTTATAGCCCTTG-3′(anti-sense). Data were normalized by the level of IgG in each sample.

### Statistical Analysis

The statistical significance of comparisons between two groups was determined with Student’s *t*-test, whereas comparisons among multiple groups were assessed by one-way analysis of variance, followed by least-significant difference -*t* test analysis, with *P* < 0.05 considered to be statistically significant.

## Additional Information

**How to cite this article**: Ren, W. *et al*. Bromodomain protein Brd3 promotes *Ifnb1* transcription via enhancing IRF3/p300 complex formation and recruitment to *Ifnb1* promoter in macrophages. *Sci. Rep.* **7**, 39986; doi: 10.1038/srep39986 (2017).

**Publisher's note:** Springer Nature remains neutral with regard to jurisdictional claims in published maps and institutional affiliations.

## Supplementary Material

Supplementary Dataset 1

## References

[b1] Akira S., Takeda K. & Kaisho T. Toll-like receptors: critical proteins linking innate and acquired immunity. Nat Immunol. 2, 675–680 (2001). 11477402 10.1038/90609

[b2] Akira S. & Takeda K. Toll-like receptor signalling. Nat Rev Immunol. 4, 499–511 (2004). 15229469 10.1038/nri1391

[b3] Honda K., Takaoka A. & Taniguchi T. Type I interferon gene induction by the interferon regulatory factor family of transcription factors. Immunity. 25, 349–360 (2006). 16979567 10.1016/j.immuni.2006.08.009

[b4] Sanchez R. & Zhou M. M. The role of human bromodomains in chromatin biology and gene transcription. Curr Opin Drug Discov Devel. 12, 659–665 (2009). PMC2921942 19736624

[b5] Mujtaba S., Zeng L. & Zhou M. M. Structure and acetyl-lysine recognition of the bromodomain. Oncogene. 26, 5521–5527 (2007). 17694091 10.1038/sj.onc.1210618

[b6] Zeng L. & Zhou M. M. Bromodomain: an acetyl-lysine binding domain. FEBS Lett. 513, 124–128 (2002). 11911891 10.1016/s0014-5793(01)03309-9

[b7] Rahman S. . The Brd4 extraterminal domain confers transcription activation independent of pTEFb by recruiting multiple proteins, including NSD3. Mol Cell Biol. 31, 2641–2652 (2011). 21555454 10.1128/MCB.01341-10 PMC3133372

[b8] Fu L. L. . Inhibition of BET bromodomains as a therapeutic strategy for cancer drug discovery. Oncotarget. 6, 5501–5516 (2015). 25849938 10.18632/oncotarget.3551 PMC4467383

[b9] Belkina A. C., Blanton W. P., Nikolajczyk B. S. & Denis G. V. The double bromodomain protein Brd2 promotes B cell expansion and mitogenesis. J Leukoc Biol. 95, 451–460 (2014). 24319289 10.1189/jlb.1112588 PMC3923082

[b10] Floyd S. R. . The bromodomain protein Brd4 insulates chromatin from DNA damage signalling. Nature. 498, 246–250 (2013). 23728299 10.1038/nature12147 PMC3683358

[b11] Wang R. . Bromodomain protein Brd4 associated with acetylated chromatin is important for maintenance of higher-order chromatin structure. J Biol Chem. 287, 10738–10752 (2012). 22334664 10.1074/jbc.M111.323493 PMC3322821

[b12] Mochizuki K. . The bromodomain protein Brd4 stimulates G1 gene transcription and promotes progression to S phase. J Biol Chem. 283, 9040–9048 (2008). 18223296 10.1074/jbc.M707603200 PMC2431025

[b13] Denis G. V. Bromodomain motifs and “scaffolding”? Front Biosci. 6, D1065–1068 (2001). 11532602 10.2741/a668 PMC3042883

[b14] LeRoy G., Rickards B. & Flint S. J. The double bromodomain proteins Brd2 and Brd3 couple histone acetylation to transcription. Mol Cell. 30, 51–60 (2008). 18406326 10.1016/j.molcel.2008.01.018 PMC2387119

[b15] Lamonica J. M. . Bromodomain protein Brd3 associates with acetylated GATA1 to promote its chromatin occupancy at erythroid target genes. Proc Natl Acad Sci USA 108, E159–168 (2011). 21536911 10.1073/pnas.1102140108 PMC3107332

[b16] Huang B. . Brd4 coactivates transcriptional activation of NF-kappaB via specific binding to acetylated RelA. Mol Cell Biol. 29, 1375–1387 (2009). 19103749 10.1128/MCB.01365-08 PMC2643823

[b17] Hargreaves D. C., Horng T. & Medzhitov R. Control of inducible gene expression by signal-dependent transcriptional elongation. Cell. 138, 129–145 (2009). 19596240 10.1016/j.cell.2009.05.047 PMC2828818

[b18] Patel M. C. . BRD4 coordinates recruitment of pause release factor P-TEFb and the pausing complex NELF/DSIF to regulate transcription elongation of interferon-stimulated genes. Mol Cell Biol. 33, 2497–2507 (2013). 23589332 10.1128/MCB.01180-12 PMC3700095

[b19] Nicodeme E. . Suppression of inflammation by a synthetic histone mimic. Nature. 468, 1119–1123 (2010). 21068722 10.1038/nature09589 PMC5415086

[b20] Chen W. . Induction of Siglec-G by RNA viruses inhibits the innate immune response by promoting RIG-I degradation. Cell. 152, 467–478 (2013). 23374343 10.1016/j.cell.2013.01.011

[b21] Filippakopoulos P. . Histone recognition and large-scale structural analysis of the human bromodomain family. Cell. 149, 214–231 (2012). 22464331 10.1016/j.cell.2012.02.013 PMC3326523

[b22] Wathelet M. G. . Virus infection induces the assembly of coordinately activated transcription factors on the IFN-beta enhancer *in vivo*. Mol Cell. 1, 507–518 (1998). 9660935 10.1016/s1097-2765(00)80051-9

[b23] Masumi A. Histone acetyltransferases as regulators of nonhistone proteins: the role of interferon regulatory factor acetylation on gene transcription. Journal of biomedicine & biotechnology. 2011, 640-610 (2011). 10.1155/2011/640610 PMC3018675 21234331

[b24] Suhara W., Yoneyama M., Kitabayashi I. & Fujita T. Direct involvement of CREB-binding protein/p300 in sequence-specific DNA binding of virus-activated interferon regulatory factor-3 holocomplex. J Biol Chem. 277, 22304–22313 (2002). 11940575 10.1074/jbc.M200192200

[b25] Yoneyama M. . Direct triggering of the type I interferon system by virus infection: activation of a transcription factor complex containing IRF-3 and CBP/p300. Embo j. 17, 1087–1095 (1998). 9463386 10.1093/emboj/17.4.1087 PMC1170457

[b26] Kanno T. . Selective recognition of acetylated histones by bromodomain proteins visualized in living cells. Mol Cell. 13, 33–43 (2004). 14731392 10.1016/s1097-2765(03)00482-9

[b27] O’Shea J. J. & Murray P. J. Cytokine signaling modules in inflammatory responses. Immunity. 28, 477–487 (2008). 18400190 10.1016/j.immuni.2008.03.002 PMC2782488

[b28] Wee S. . Targeting epigenetic regulators for cancer therapy. Ann N Y Acad Sci. 1309, 30–36 (2014). 24571255 10.1111/nyas.12356

[b29] Liu X. . CaMKII promotes TLR-triggered proinflammatory cytokine and type I interferon production by directly binding and activating TAK1 and IRF3 in macrophages. Blood. 112, 4961–4970 (2008). 18818394 10.1182/blood-2008-03-144022

[b30] Liu X. . Intracellular MHC class II molecules promote TLR-triggered innate immune responses by maintaining activation of the kinase Btk. Nat Immunol. 12, 416–424 (2011). 21441935 10.1038/ni.2015

[b31] An H. . Src homology 2 domain-containing inositol-5-phosphatase 1 (SHIP1) negatively regulates TLR4-mediated LPS response primarily through a phosphatase activity- and PI-3K-independent mechanism. Blood. 105, 4685–4692 (2005). 15701712 10.1182/blood-2005-01-0191

[b32] Wang C. . Zinc finger protein 64 promotes Toll-like receptor-triggered proinflammatory and type I interferon production in macrophages by enhancing p65 subunit activation. J Biol Chem. 288, 24600–24608 (2013). 23857586 10.1074/jbc.M113.473397 PMC3750158

[b33] Wu Y. . Expression of CD40 and growth-inhibitory activity of CD40 ligand in colon cancer *ex vivo*. Cellular immunology. 253, 102–109 (2008). 18603231 10.1016/j.cellimm.2008.05.005

