# Author Correction: Bromodomain protein Brd3 promotes *Ifnb1* transcription via enhancing IRF3/p300 complex formation and recruitment to *Ifnb1* promoter in macrophages

**DOI:** 10.1038/s41598-020-77134-7

**Published:** 2020-12-01

**Authors:** Wenhui Ren, Chunmei Wang, Qinlan Wang, Dezhi Zhao, Kai Zhao, Donghao Sun, Xingguang Liu, Chaofeng Han, Jin Hou, Xia Li, Qian Zhang, Xuetao Cao, Nan Li

**Affiliations:** 1 National Key Laboratory of Medical Immunology and Institute of Immunology, Second Military Medical University, Shanghai, 200433 China; 2 Institute of Basic Medical Sciences, Chinese Academy of Medical Sciences, School of Basic Medicine Peking Union Medical College, Beijing, 100005 China; 3 Institute of Immunology, Zhejiang University School of Medicine, Hangzhou, 310058 Zhejiang China

Correction to: *Scientific Reports* 10.1038/srep39986, published online 3 January 2017

The data provided in this Article is incomplete.

Raw blotting data was not included in the original published. It is now included in the Supplement File included with this notice. However, data for the vsv-actin, sev-brd3, and sev-actin blots of Figure 1C, and the wcl-irf3 and wcl-actin blots of Figure 3D, could not be retrieved due to the time that has elapsed since the conclusion of the project.

## Supplementary information

Supplementary Information.

