# Supplementary material for: Bromodomain protein Brd3 promotes Ifnb1 transcription via enhancing IRF3/p300 complex formation and recruitment to Ifnb1 promoter in macrophages
Source: Sci Rep. 2017 Jan 3;7:39986. doi: 10.1038/srep39986 (PMC5206742; doi:10.1038/srep39986)

**Bromodomain protein Brd3 promotes *Ifnb1* transcription via enhancing IRF3/p300 complex formation and recruitment to *Ifnb1* promoter in macrophages**

Wenhui Ren, Chunmei Wang, Qinlan Wang, Dezhi Zhao, Kai Zhao, Donghao Sun, Xingguang Liu, Chaofeng Han, Jin Hou, Xia Li, Qian Zhang, Xuetao Cao, Nan Li

**Supplementary figure legends**

**Supplementary Figure 1.** The effects of Brd3 to the TNF-α and IL-6 production after virus infection. (a), Western blots with antibody against Brd3 protein to screen the cells having Brd3 knocked out using CRISPR-Cas9 technology in RAW264.7 cells. (b), Brd3-ko cells and control cells were infected with HSV (MOI=10), VSV(MOI=10) or SeV(MOI=10) for 12 hours or left unstimulated (Med), and then the production of IL-6 in the supernatants was measured by ELISA. (c), Brd3-ko cells and control cells were infected with HSV (MOI=10) or VSV(MOI=10) for 12 hours or left unstimulated (Med), and then the production of TNF-α in the supernatants was measured by ELISA. Data are shown as means±SD of three independent experiments. ＊*P*<0.05(Student’s *t*-test).

**Supplementary Figure 2.** Brd3 promotes *Ifnb1* and *Il6* expression in RAW264.7 cells. (a), Two different clones of Brd3-ko cells(Brd3-ko-1 and Brd3-ko-2) and control cells were infected with HSV (MOI=10), VSV(MOI=10) or SeV(MOI=10) for the indicated times. The *Ifnb1* mRNA level was detected by Q-PCR, the results were presented as fold expression of *Ifnb1* mRNA to that of *Actb*. (b), The cells were treated as in a, the *Il6* mRNA level was detected by Q-PCR, the results were presented as fold expression of *Il6* mRNA to that of *Actb*. (c), The cells were treated as in a, the *Tnfa* mRNA level was detected by Q-PCR, the results were presented as fold expression of *Tnfa* mRNA to that of *Actb*. (d), The cells were infected with VSV(MOI=10) for the indicated times, the VSV mRNA level was detected by Q-PCR and normalized to that of *Actb*, the results were presented as ‘fold induction’ relative to expression in control cells without VSV infection*.* Data are shown as means±SD of three independent experiments. ＊*P*<0.05(ANOVA).

**Supplementary Figure 3.** Brd3 promotes *Ifnb1* and *Il6* expression in macrophages. (a), Mouse primary peritoneal macrophages were transiently transfected with si-RNA targeting Brd3. 24 hours later, the *Brd3* mRNA expression level was detected by Q-PCR. The results were presented as fold expression of *Brd3* mRNA to that of *Actb*. (b), Mouse primary peritoneal macrophages were transiently transfected with si-Brd3 or si-Non, 24 hours later, the cells were infected with HSV (MOI=10) or VSV(MOI=10) for the indicated times. The *Ifnb1* mRNA level was detected by Q-PCR, the results were presented as fold expression of *Ifnb1* mRNA to that of *Actb*. (c), The cells were treated as in a, the *Il6* mRNA level was detected by Q-PCR, the results were presented as fold expression of *Il6* mRNA to that of *Actb*. Data are shown as means±SD of three independent experiments. ＊*P*<0.05(Student’s *t*-test).

**Supplementary Figure 4.** Brd3 re-expressed in Brd3-ko cells. Brd3-ko cells were transiently transfected with a Brd3 expression plasmid (Brd3-Myc) or empty vector, 24 hours later, the expression of Myc-tagged Brd3 was detected by Western blot analysis.

**Supplementary Figure 5.** Brd3 associates with acetyl-histone3 and acetyl-histone4. RAW264.7 cells were lysed and immunoprecipitated (500μg) with Brd3 antibody and then immunoblotted with the indicated antibodies. Similar results were obtained in three independent experiments.

**Supplementary Figure 6.** Brd3 has no effect of the acetylation level of histone3/4 within *Tnfa* gene promoter. Brd3-ko cells and control cells were infected with VSV (MOI=10) for 8 hours or left unstimulated (Med). The amount of acetylated histone3 and histone4 within *Tnfa* gene promoter was detected with a ChIP assay and analyzed by Q-PCR. Data are shown as means±SD of three independent experiments. ＊*P*<0.05(Student’s *t*-test).

**Supplementary figure 1**


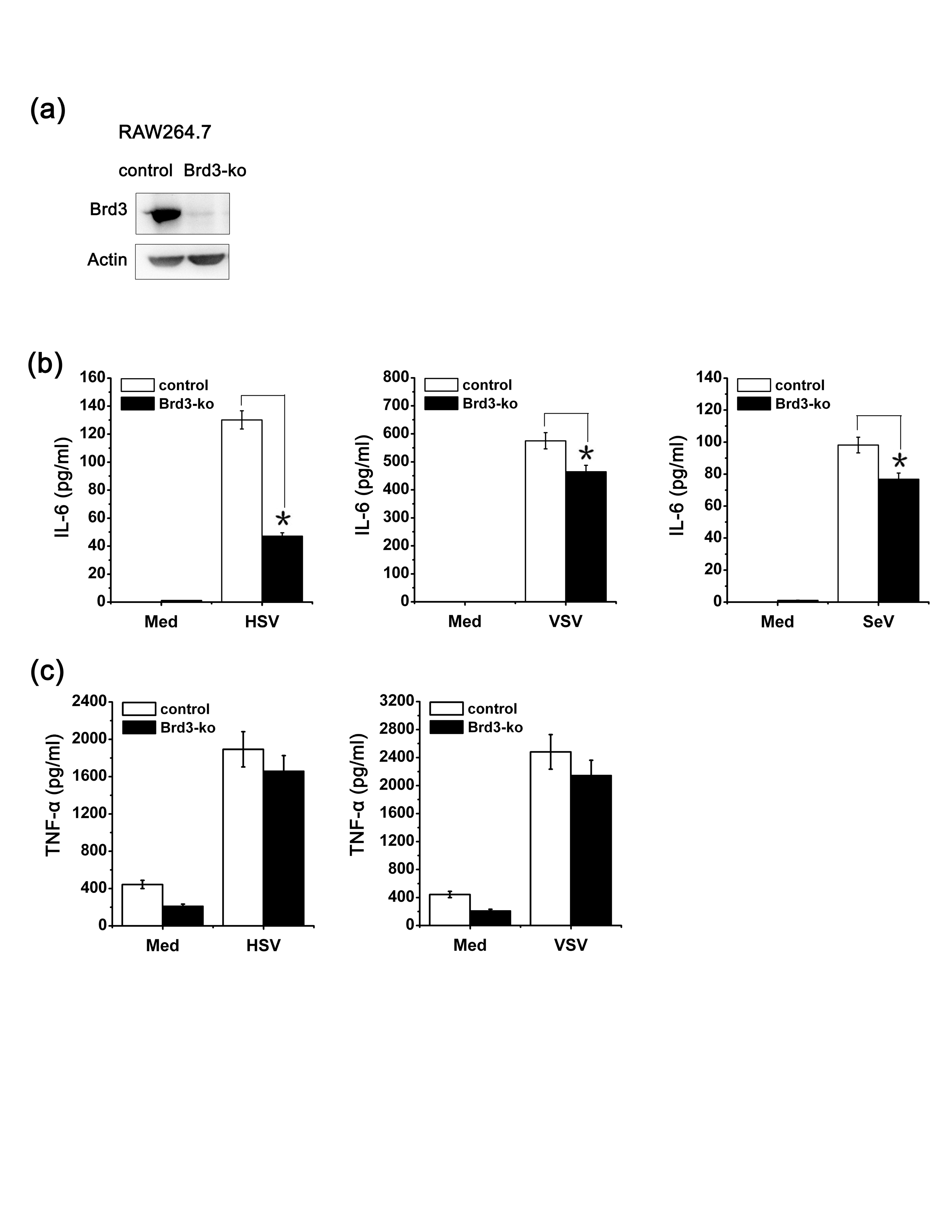


**Supplementary figure 2**


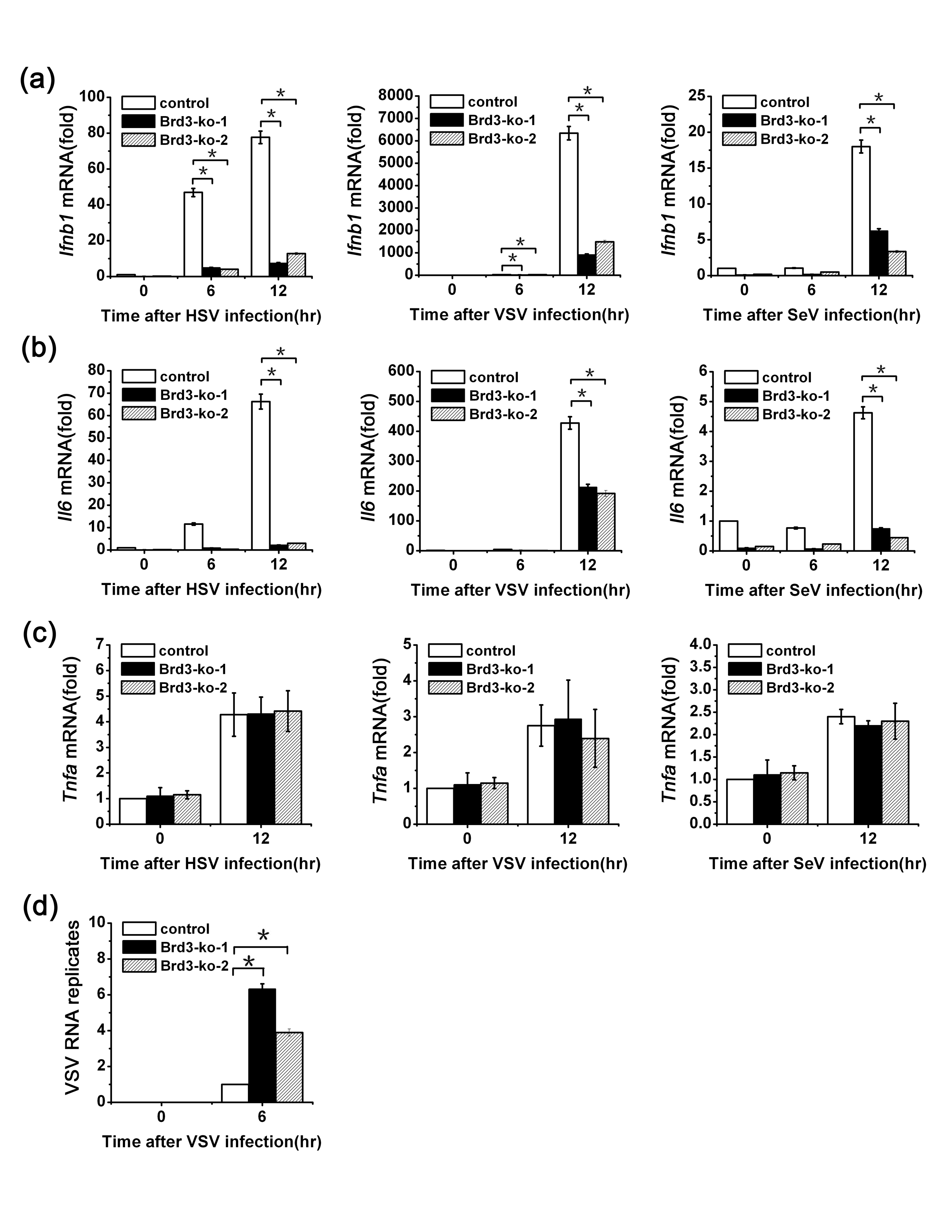


**Supplementary figure 3**


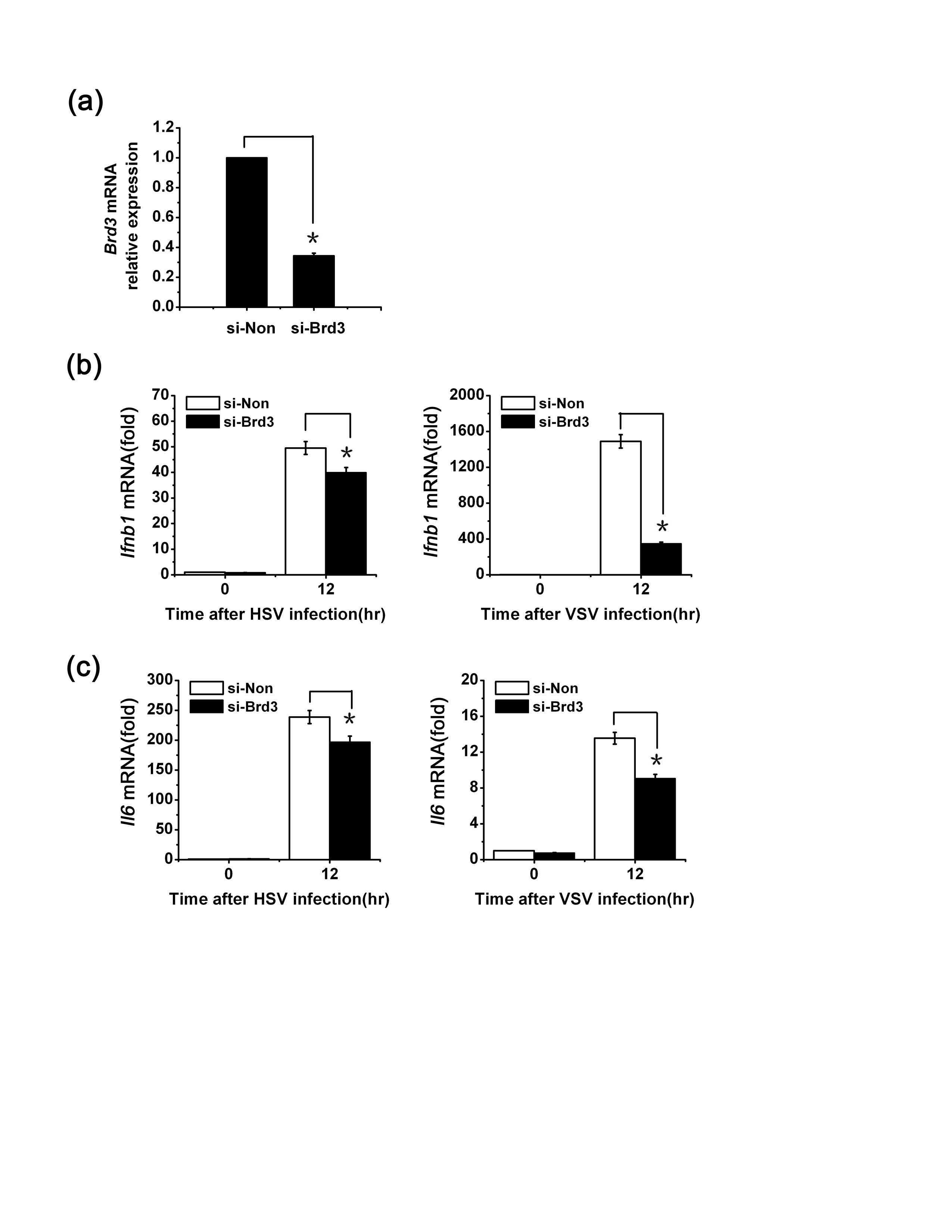


**Supplementary figure 4**


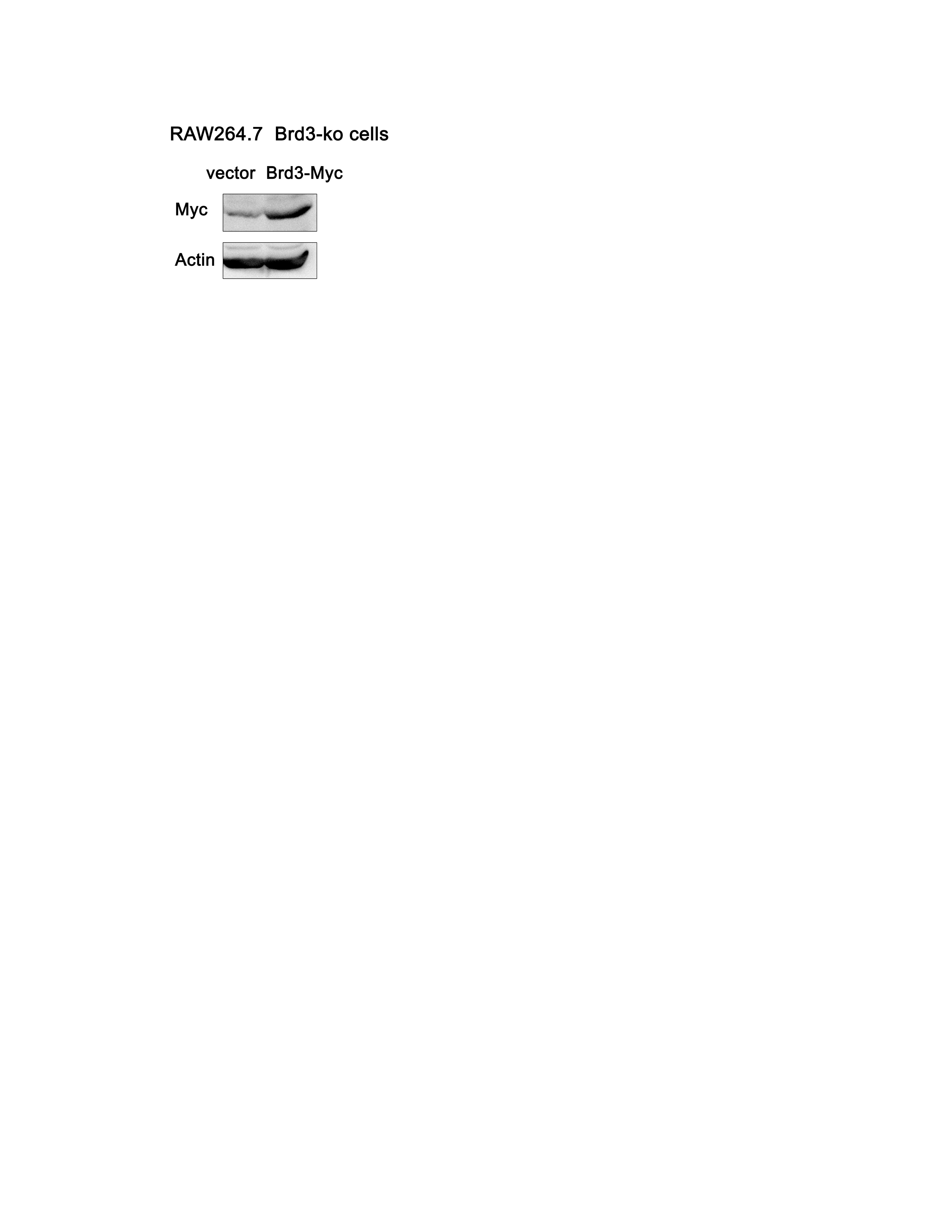


**Supplementary figure 5**


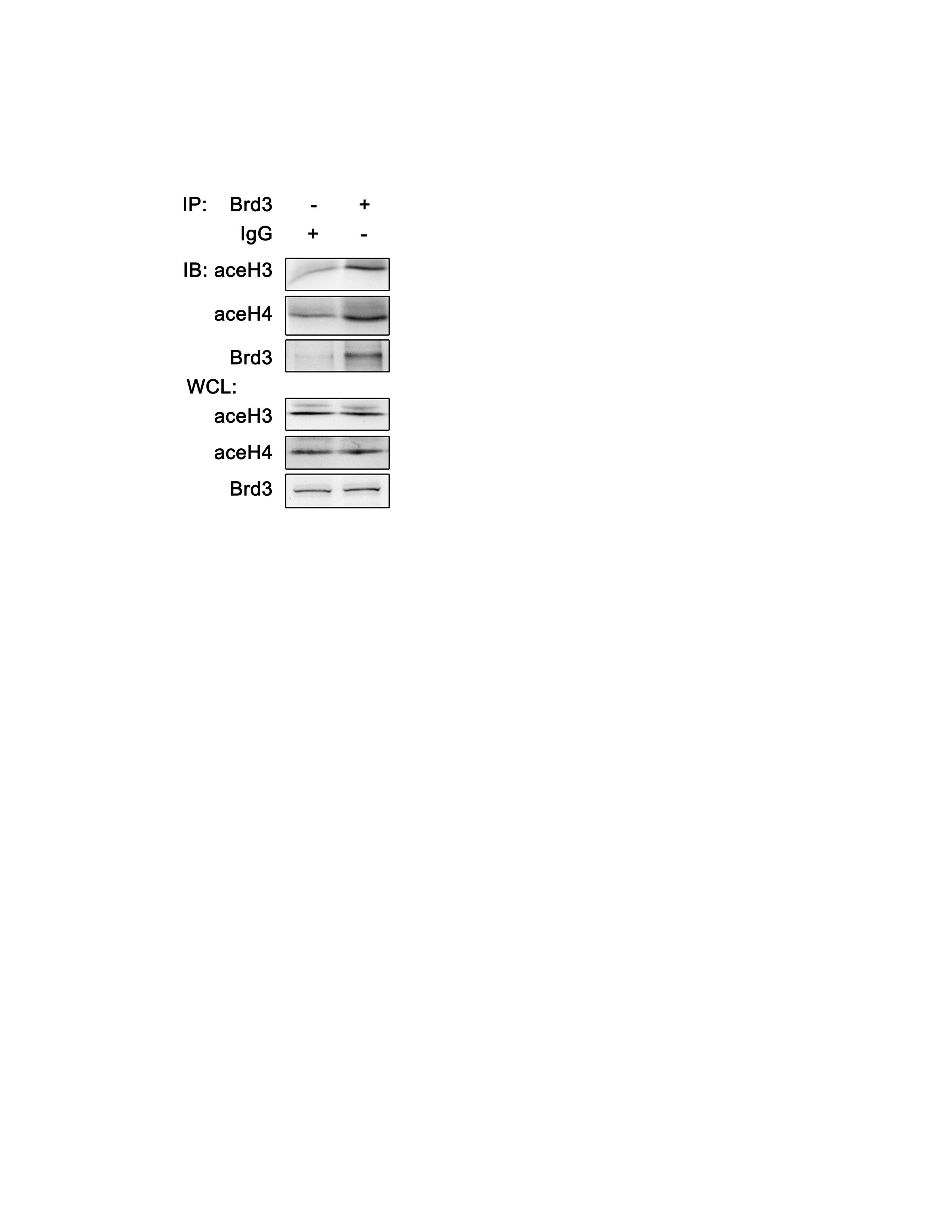


**Supplementary figure 6**


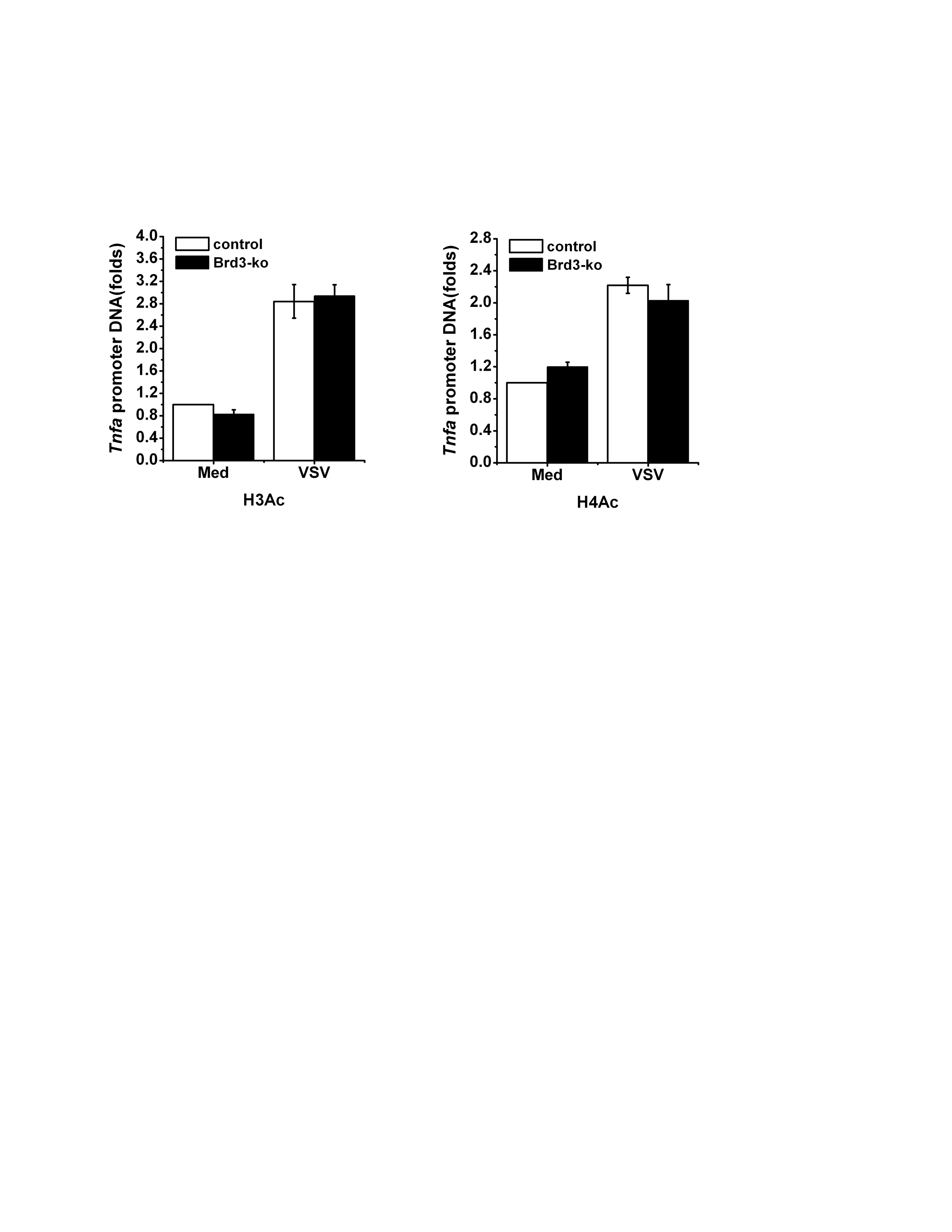

Supplement: Supplementary Dataset 1 [file srep39986-s1.doc]
