# Supplementary material for: Author Correction: Bromodomain protein Brd3 promotes Ifnb1 transcription via enhancing IRF3/p300 complex formation and recruitment to Ifnb1 promoter in macrophages
Source: Sci Rep. 2020 Dec 1;10:21363. doi: 10.1038/s41598-020-77134-7 (PMC7708833; doi:10.1038/s41598-020-77134-7)
Supplement: Supplementary file 1 — Supplementary Information. [file 41598_2020_77134_MOESM1_ESM.pdf]

Bromodomain protein Brd3 promotes *Ifnb1* transcription via enhancing IRF3/p300 complex formation and recruitment to *Ifnb1* promoter in macrophages

Wenhui Ren[1*], Chunmei Wang[2*], Qinlan Wang[3*], Dezhi Zhao[3], Kai Zhao[2], Donghao Sun[2], Xingguang Liu[1], Chaofeng Han[1], Jin Hou[1], Xia Li[2], Qian Zhang[1], Xuetao Cao[1,2] & Nan Li[1]

[1]National Key Laboratory of Medical Immunology and Institute of Immunology, Second Military Medical University, Shanghai 200433

[2]Institute of Basic Medical Sciences, Chinese Academy of Medical Sciences, School of Basic Medicine Peking Union Medical College, Beijing 100005

[3]Institute of Immunology, Zhejiang University School of Medicine, Hangzhou 310058, Zhejiang, China

[*]These authors contributed equally to this work.

Correspondence and requests for materials should be addressed to N.L.(email: linan@immunol.org)

Uncropped data for Figure1

Figure 1a

Brd3

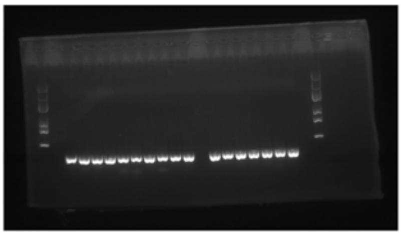

Actb

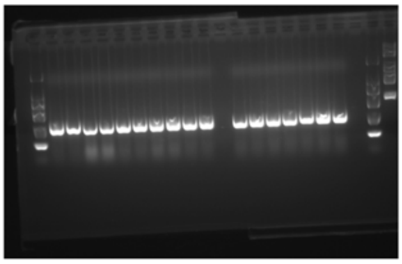

Figure 1c

HSV

Brd3

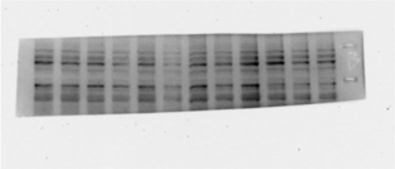

Actin

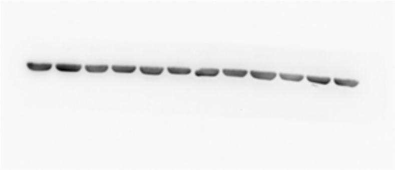

VSV

Brd3

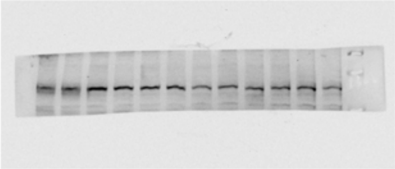

Uncropped data for Figure3

Figure 3a

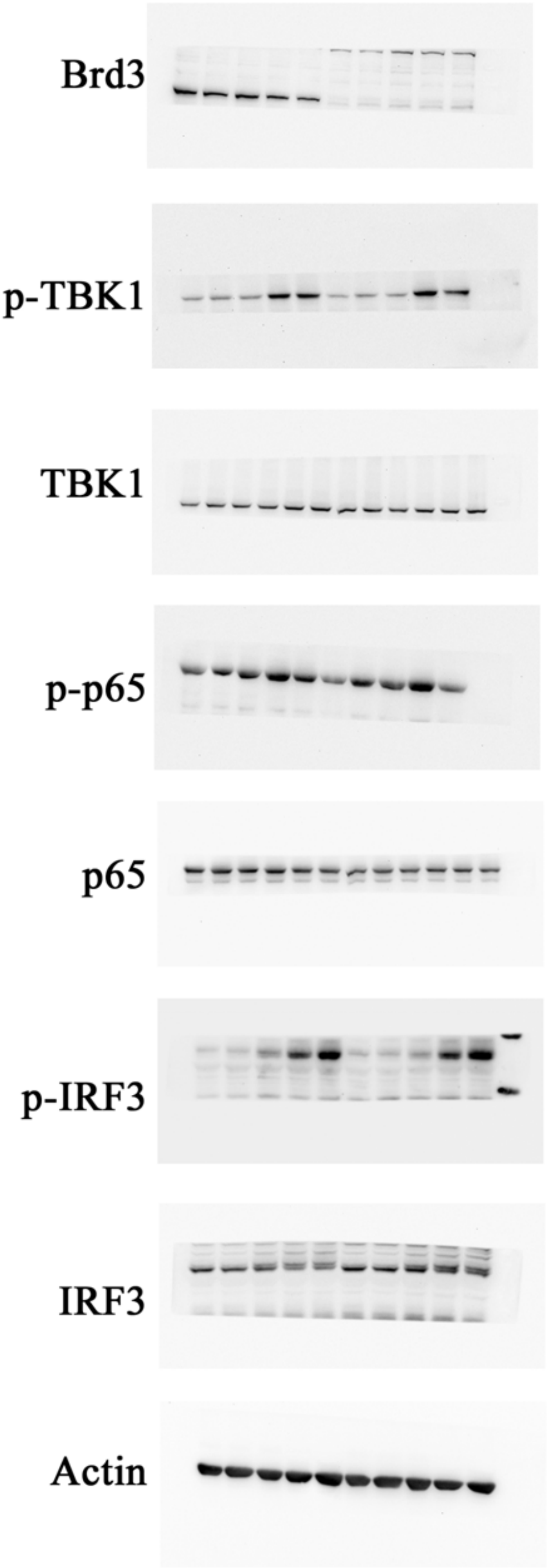

Figure 3b

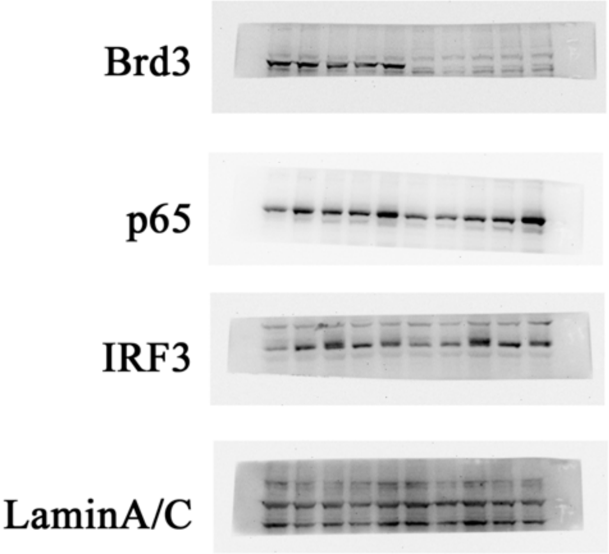

Figure 3c

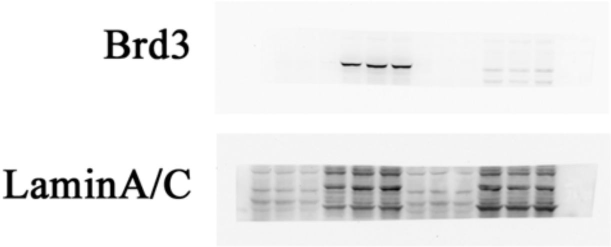

Figure 3d

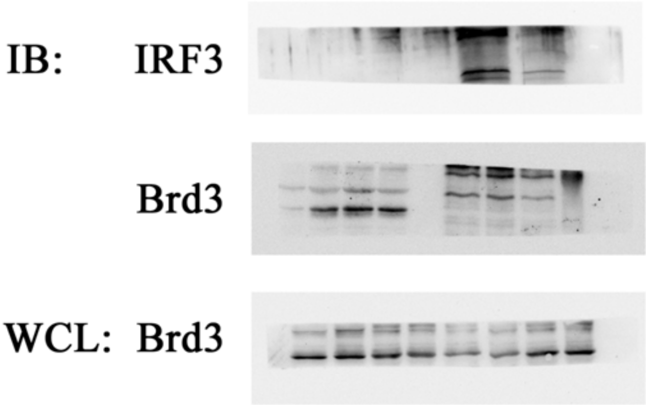

Uncropped data for Figure4

Figure 4a

IB: aceK

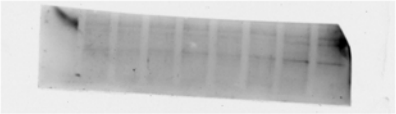

CBP

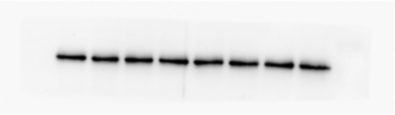

WCL: Brd3

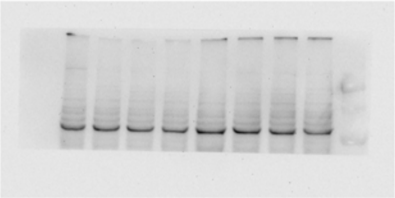

CBP

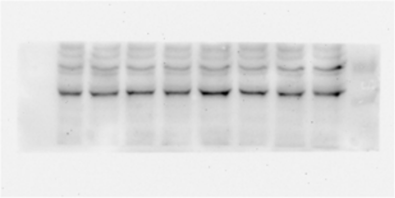

Actin

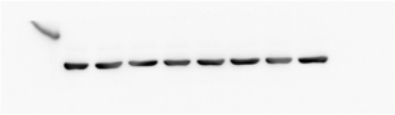

Figure 4b

IB: aceK

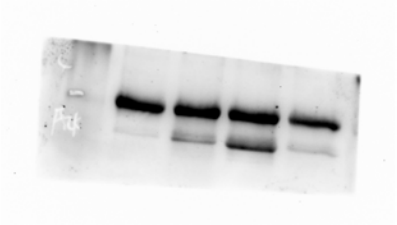

FLAG

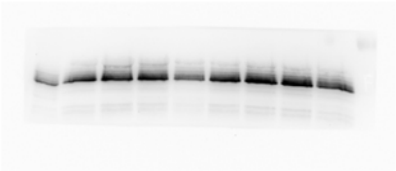

WCL: Myc

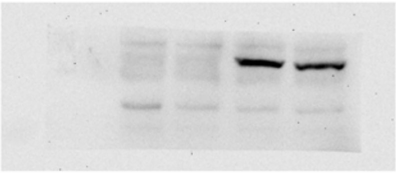

FLAG

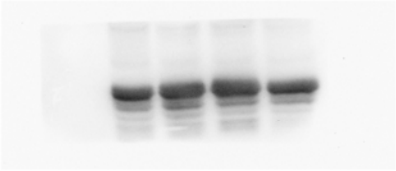

Uncropped data for Figure5

Figure 5a

IB: p300

Brd3

WCL: Brd3

p300

Actin

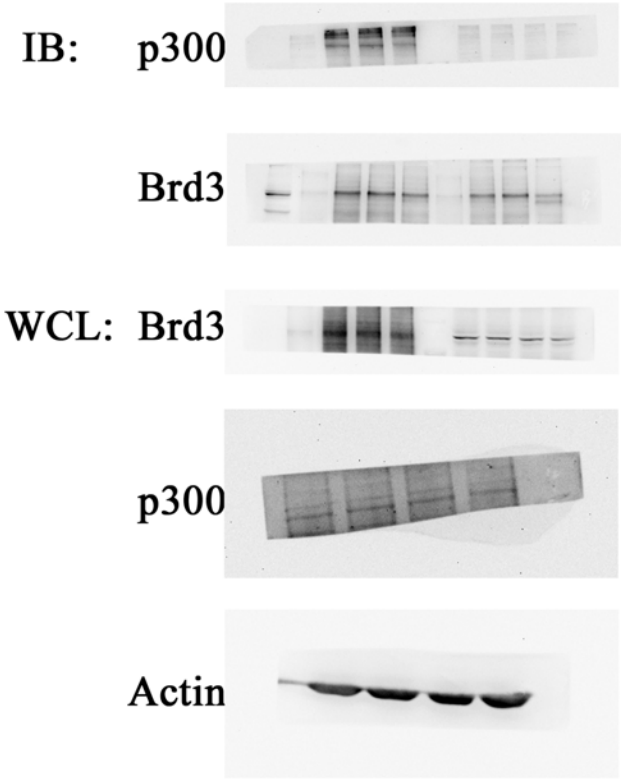

Figure 5b

IB: p300

IRF3

WCL: p300

Brd3

IRF3

LaminA/C

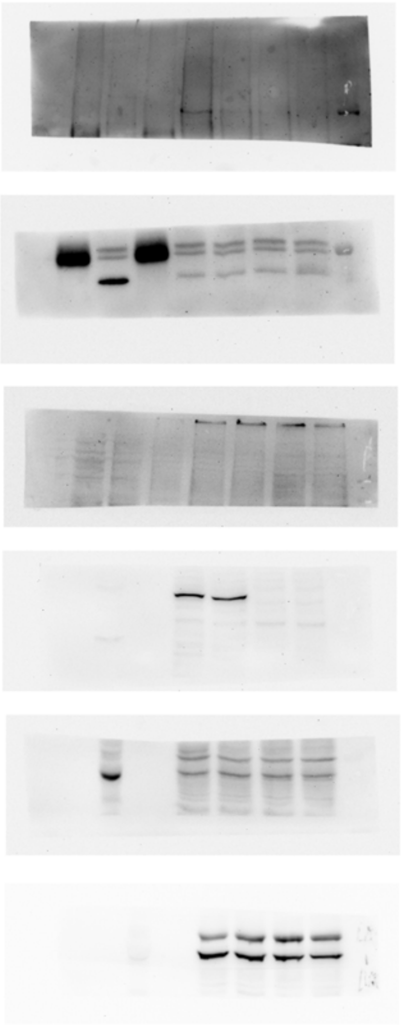

Figure 5c

IB: p300

FLAG

WCL: Myc

FLAG

Actin

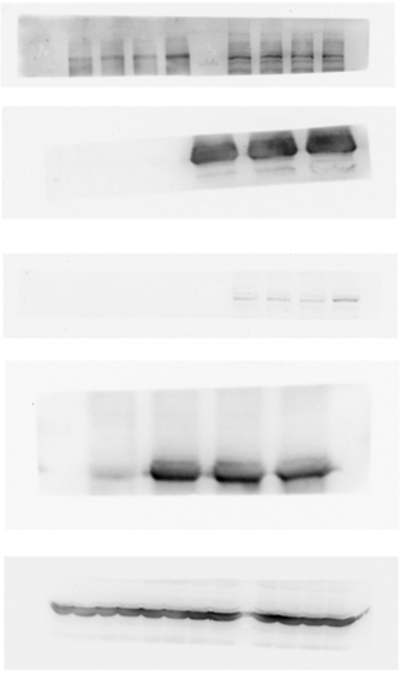

Uncropped data for supplymentary data

S.Fig. 1a

Brd3

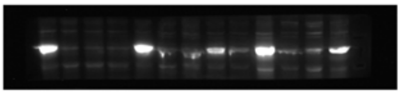

Actin

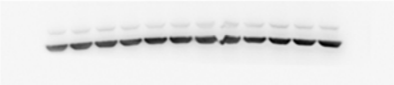

S.Fig. 4

Myc

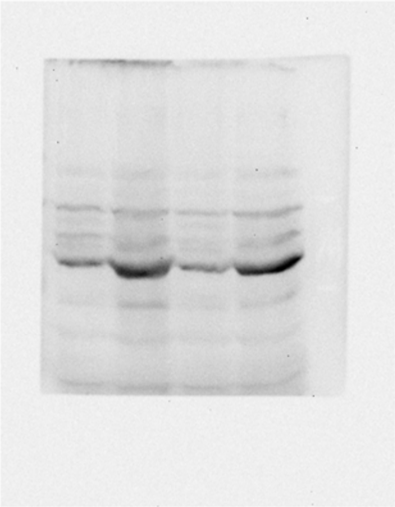

Actin

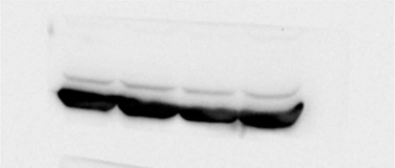

S.Fig. 5

IB: aceH3

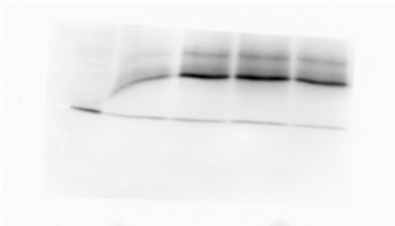

aceH4

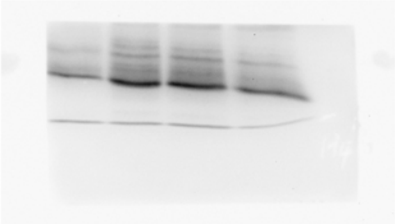

Brd3

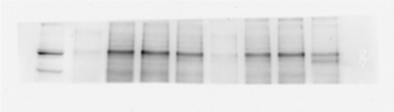

WCL: aceH3

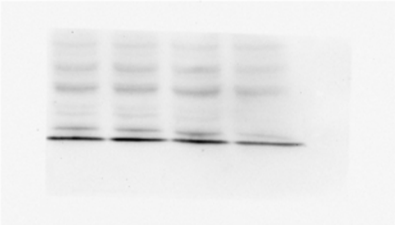

aceH4

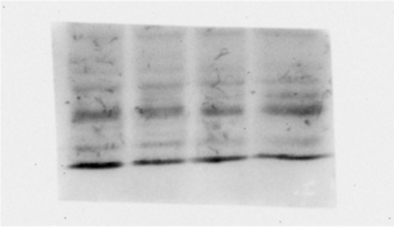

Brd3
